# Granular activated carbon enhances volatile fatty acid production in the anaerobic fermentation of garden wastes

**DOI:** 10.3389/fbioe.2023.1330293

**Published:** 2023-12-11

**Authors:** Wenwen Chen, Yiwei Zeng, Huanying Liu, Dezhi Sun, Xinying Liu, Haiyu Xu, Hongbin Wu, Bin Qiu, Yan Dang

**Affiliations:** ^1^ Beijing Key Laboratory for Source Control Technology of Water Pollution, Engineering Research Center for Water Pollution Source Control and Eco-Remediation, College of Environmental Science and Engineering, Beijing Forestry University, Beijing, China; ^2^ Qinglin Chuangneng (Shanghai) Technology Co., Ltd., Shanghai, China

**Keywords:** anaerobic fermentation, garden waste, VFAs, GAC, microbial community, metagenomics analysis

## Abstract

Garden waste, one type of lignocellulosic biomass, holds significant potential for the production of volatile fatty acids (VFAs) through anaerobic fermentation. However, the hydrolysis efficiency of garden waste is limited by the inherent recalcitrance, which further influences VFA production. Granular activated carbon (GAC) could promote hydrolysis and acidogenesis efficiency during anaerobic fermentation. This study developed a strategy to use GAC to enhance the anaerobic fermentation of garden waste without any complex pretreatments and extra enzymes. The results showed that GAC addition could improve VFA production, especially acetate, and reach the maximum total VFA yield of 191.55 mg/g VS_added_, which increased by 27.35% compared to the control group. The highest VFA/sCOD value of 70.01% was attained in the GAC-amended group, whereas the control group only reached 49.35%, indicating a better hydrolysis and acidogenesis capacity attributed to the addition of GAC. Microbial community results revealed that GAC addition promoted the enrichment of *Caproiciproducens* and *Clostridium*, which are crucial for anaerobic VFA production. In addition, only the GAC-amended group showed the presence of *Sphaerochaeta* and *Oscillibacter* genera, which are associated with electron transfer processes. Metagenomics analysis indicated that GAC addition improved the abundance of glycoside hydrolases (GHs) and key functional enzymes related to hydrolysis and acidogenesis. Furthermore, the assessment of major genera influencing functional genes in both groups indicated that *Sphaerochaeta*, *Clostridium*, and *Caproicibacter* were the primary contributors to upregulated genes. These findings underscored the significance of employing GAC to enhance the anaerobic fermentation of garden waste, offering a promising approach for sustainable biomass conversion and VFA production.

## 1 Introduction

Currently, the critical issue of energy shortage has drawn increasing attention toward the quest for alternative fossil fuels and the exploration of emerging green and sustainable technologies. Garden waste, a form of lignocellulosic biomass, comprises various organic materials (e.g., grass and flower cuttings, hedge trimmings, tree pruning, small branches, fallen leaves, and wood debris) (ten Hoeve et al., 2019; Li et al., 2020). The pruning of green leaves possesses a significant amount of organic matter and is abundant in quantity. In comparison to other garden wastes, leaves exhibit a relatively low lignin content, making them highly potential for energy conversion (Ramprakash and Incharoensakdi, 2022). The intricate composition of garden waste offers the potential for transformation into a variety of biofuels and intermediate chemicals, thereby producing high-value products (Ren et al., 2016).

In recent years, the anaerobic digestion of lignocellulosic biomass has been increasingly developed, compared to ethanol and other alcohols, carboxylic acids are thermodynamically favored when methanogenesis is inhibited during anaerobic digestion and could still achieve high product yields without sterile conditions (Darvekar et al., 2019). Volatile fatty acids (VFAs) have attracted attention from many researchers due to their widespread applications across the industries, such as food, pharmaceuticals, chemicals, agriculture, and wastewater treatment (Soares et al., 2010; Ramos-Suarez et al., 2021). VFAs exhibit significant potential for conversion into green chemicals and as substitutes for fossil fuels. Hence, the production of high-value VFAs by anaerobic fermentation emerges as a viable alternative to anaerobic digestion.

However, the composition of lignocellulosic biomass, particularly comprising cellulose, hemicellulose, and lignin, results in a highly resistant and recalcitrant structure due to the interactions between these components. As a consequence, the hydrolysis of lignocellulose is usually the rate-limiting step considered during traditional anaerobic digestion (Sawatdeenarunat et al., 2015). Therefore, the low hydrolysis efficiency of garden waste in the anaerobic fermentation process leads to a large amount of incompletely treated waste, which decreases the resource utilization efficiency. In this regard, various pretreatment techniques, including chemical, physical, biological, and their combinations, have been developed to improve the efficiency (Zhao et al., 2022; Fang et al., 2023). Nonetheless, these pretreatment methods suffer from a range of drawbacks, such as complex operational processes, high costs, secondary pollution, high energy consumption, and possibly other issues as well (Fonoll et al., 2021; Mankar et al., 2021). Given the above considerations, it is essential to find an eco-friendly approach to enhance the conversion efficiency of garden waste and VFA production during anaerobic fermentation, without resorting to conventional sophisticated pretreatment processes (Cao et al., 2023).

Granular activated carbon (GAC), serving as a conductive material with a notably specific surface area, provides additional attachment sites for microbes. This shortens the distance between syntrophic partners and enhances mass transfer (Li et al., 2021), leading to elevated biofilm formation and improved the yield of the target products (Ma et al., 2022). GAC facilitates electron transfer, modulates metabolic pathways, and enables the formation of an electron transfer chain, promoting electron sharing and interactions among bacteria (Lovley, 2017; Yang et al., 2017; Zhang et al., 2017). This optimization further optimizes the hydrolytic fermentation and acid production process. Presently, the majority of research on the anaerobic fermentation of lignocellulosic biomass primarily emphasizes pretreatment techniques (Kumar et al., 2022). There have been no studies that have investigated the impact of adding GAC on both the VFA production and microorganisms in lignocellulosic biomass without employing any complex pretreatments or enzymes.

Therefore, batch tests were conducted in this study with the same pruning of green leave concentrations to clarify the role of GAC in enhancing anaerobic fermentation. The main objective of this research was to examine whether the inclusion of GAC could enhance the efficiency of anaerobic fermentation in treating garden waste, eliminating the need for additional enzymes or complex pretreatment processes. Furthermore, the impact of GAC on VFA production and the underlying mechanisms were explored through 16S rRNA gene and metagenomics analysis. These findings shed new light on the anaerobic fermentation of lignocellulosic biomass for VFA production, providing an approach that avoids complex pretreatment operations and high costs while promoting resource utilization and minimizing environmental pollution.

## 2 Materials and methods

### 2.1 Substrate and inoculum

In this particular investigation, garden waste comprises the discarded pruning green leaves collected from lawnmower clippings in Beijing Forestry University, China, 40.005875°N 116.347459°E. The leaves were dried at 60°C for 12 h before being ground to a particle size of less than 1 mm using a laboratory high-speed multifunctional mill (CHAORAN, CR-100, China). The ground material was sieved through a 40–80 mesh (Tyler Standard Screen Scale). Subsequently, particles in the range of 0.180–0.425 mm were carefully chosen. Afterward, the large particles were collected and ground again to obtain the desired size, and they were then sealed in plastic bags and stored at ambient temperature before conducting experiments. Anaerobic sludge was obtained from a full-scale up-flow anaerobic sludge bed (UASB) reactor located in Beijing, China, and stored at 4°C, which was broken up before being utilized as inoculum for anaerobic tests. Table 1 shows the characteristics of both the garden waste and inoculum utilized in this study.

**TABLE 1:** Characteristics of garden waste and the inoculum.

	pH	Total solid (TS) (%)	Volatile solid (VS) (%)	COD (mg/L)	Cellulose (%)	Hemicellulose (%)	Lignin (%)
Garden waste	—	99.16 ± 0.06	88.86 ± 0.18	—	12.60 ± 0.18	11.85 ± 0.66	18.12 ± 0.19
Inoculum	7.66 ± 0.02	4.94 ± 0.07	3.74 ± 0.06	304.5 ± 4.95	—	—	—

### 2.2 Reactor set-up and operation conditions

Two batch experiments for anaerobic fermentation were carried out using 1-L glass serum bottles with a working volume of 0.7 L, both bottles containing 250 mL of anaerobic sludge, 10% total solids (TS) of garden waste, and 15 mM sodium 2-bromoethanesulfonate (BES) to inhibit methane production for VFA accumulation (Chidthaisong and Conrad, 2000). One of the reactors was supplemented with 50 g/L of GAC, characterized by a particle size of 8–20 mesh, a volume of approximately 120 cm^3^, and a geometric surface area of approximately 900 cm^2^. Prior to commencing the experiment, carbon dioxide gas was employed to purge both test bottles for 10 min, displacing the air and establishing an anaerobic environment. Subsequently, both bottles were placed in a shaking incubator set to mesophilic temperature (35°C) and operated at 120 rpm. A gas-sampling bag was affixed to the outlets situated at the top of both reactors. The reactors were running for a total of 28 days, and a schematic diagram of these reactors is presented in Supplementary (Supplementary Figure S1).

### 2.3 Analytical methods

The composite samples were retrieved from the serum bottles at predetermined intervals and subsequently centrifuged at 10,000 rpm for 5 min. The supernatant was filtered through a 0.45-μm membrane for the subsequent analysis of soluble chemical oxygen demand (sCOD) and filtered through a 0.22-μm membrane for the analysis of VFAs.

The COD, TS, and volatile solids (VS) were determined with standard methods (APHA, 2005). Acetate, propionate, butyrate, valerate, and caproate were measured by high-performance liquid chromatography (HPLC) (Bio-Rad, Hercules, California) using 5 mM H_2_SO_4_ as the mobile phase (Huang et al., 2022). The cumulative VFAs, calculated as acetate (g/L), were the sum of acetate, propionate, isobutyrate, and n-butyrate during fermentation. pH was measured with a pH meter (HACH, United States). The gas volume collected in a 1-L sampling bag was measured using an air pump every 1–3 days, and then, the gas was transferred back into the bag. The hydrogen content in both the gaseous samples in the sampling bag and the headspace was measured using gas chromatography apparatus equipped with a thermal conductivity detector (TCD) (Tianmei, GC7900, China). In addition, the decomposition of garden waste was assessed by Fourier-transform infrared (FT-IR) spectroscopy (Bruker; VERTEX 70; Germany) through a full scan across the wavenumber range of 400–4,000 cm^−1^.

### 2.4 Microbial community analysis—16S rRNA gene

DNA was extracted from 2 mL sludge samples collected from both reactors at the end of the day (on day 28) using the RNeasy PowerSoil DNA Elution kit, according to the manufacturer’s instructions. The 16S rRNA gene fragments from extracted DNA samples were amplified via the polymerase chain reaction (PCR) with universal primer sets (338F/806R). Amplicons were sequenced on an Illumina HiSeq 2000 platform (Illumina, San Diego, United States) by Majorbio Bio-Pharm Technology Co. Ltd. (Shanghai, China). Subsequently, the sequences were categorized into different operational taxonomic units using Pyrosequencing Pipeline software (https://pyro.cme.msu.edu).

### 2.5 Metagenomics analysis

The specific operation of metagenomics analysis is shown in Supplementary Material.

## 3 Results and discussion

### 3.1 Performance of batch reactors

**FIGURE 1:**
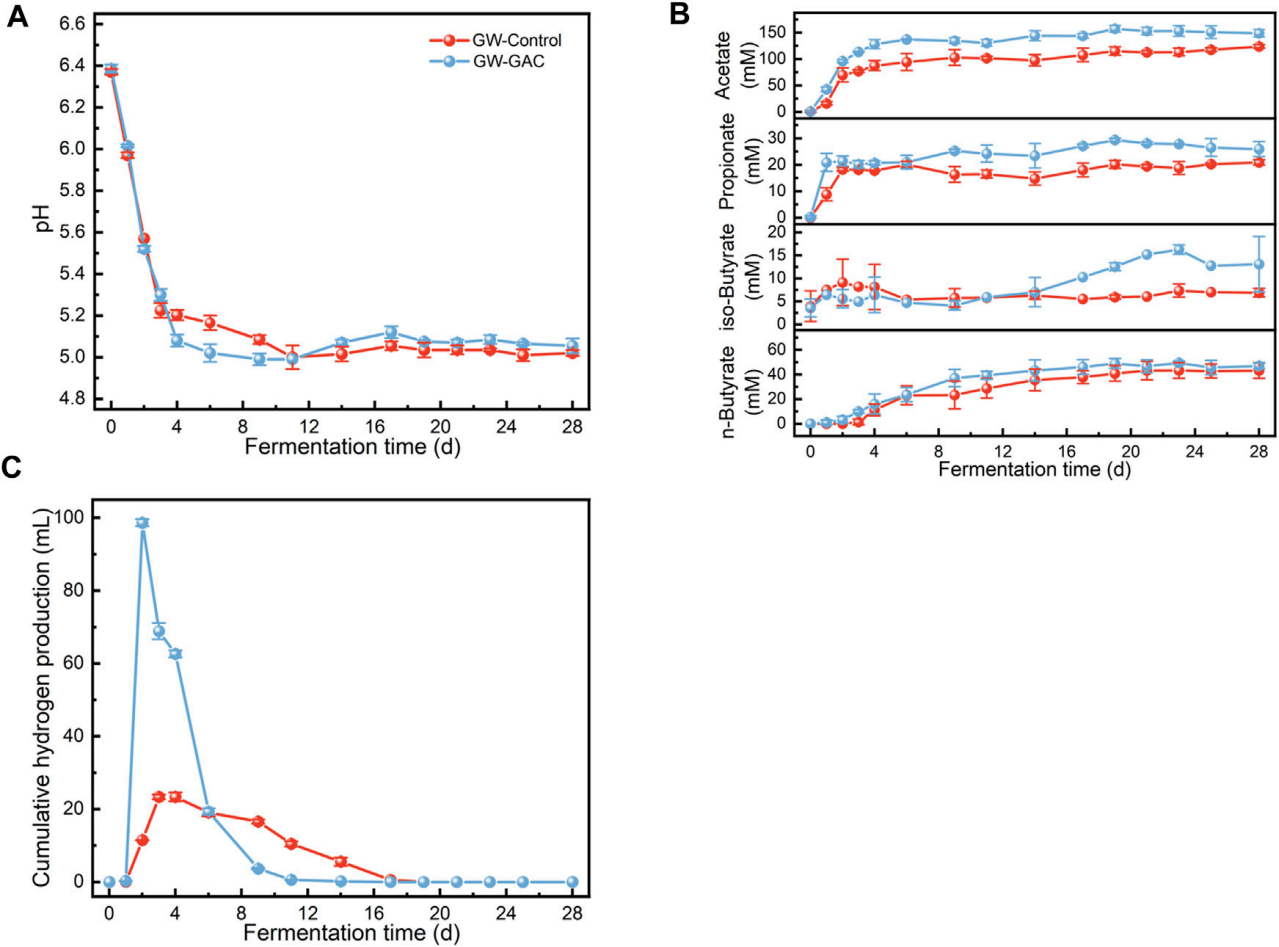
Variation in **(A)** pH, **(B)** acetate, propionate, iso-butyrate, and n-butyrate concentration, and **(C)** hydrogen accumulation in GW-GAC and GW-Control reactors during anaerobic fermentation.

The pH values of both reactors were determined, and Figure 1A shows the pH variation during the fermentation period. Both reactors exhibited a similar trend, with pH values rapidly decreasing in the initial 4 days and the GAC group experiencing a faster decline. However, from day 4 to day 28 of fermentation, no significant changes in pH values were observed in either group. The pH remained relatively stable at approximately 5.0, indicating that the activity of acidogenic bacteria was inhibited by low pH, leading to the suppression of accumulated VFA production.

Both reactors had the similar VFA compositions, and the concentrations of acetate, propionate, isobutyrate, and n-butyrate in the GAC-amended and non-amended reactors are shown in Figure 1B. Notably, no valerate or caporate was detected. The acetate concentration in the GAC-amended reactor rapidly increased in the initial 4 days, reaching a maximum concentration of 157.41 mM. On the first day, the acetate concentration in the GAC-amended reactor was approximately 2.66 times higher (42.83 mM) than that in the control group (16.09 mM), highlighting GAC’s ability to expedite acetate production. Overall, the GAC-amended reactor maintained a higher efficiency, as reflected by the higher concentrations of each VFA, particularly acetate. The crucial raw components, such as acetate, propionate, isobutyrate, and n-butyrate, used for synthesizing various valuable compounds (Xu et al., 2023), constituted the primary components of VFAs. In the GW-GAC group, these components averaged 67.01%, 13.40%, 4.62%, and 14.97%, respectively. Therefore, utilizing GAC to improve the production of acetate, propionate, and butyrate from lignocellulosic biomass like garden waste is a sustainable development route. Furthermore, cumulative hydrogen was measured, revealing an initial increase followed by gradual consumption (Figure 1C). In the GW-GAC group, the highest cumulative hydrogen production was 98.65 mL, which was 4.23 times higher than the highest cumulative hydrogen production in the GW-Control group (23.35 mL). These findings may suggest that adding GAC increased the abundance of crucial functional enzymes in the anaerobic fermentation system.

**FIGURE 2:**
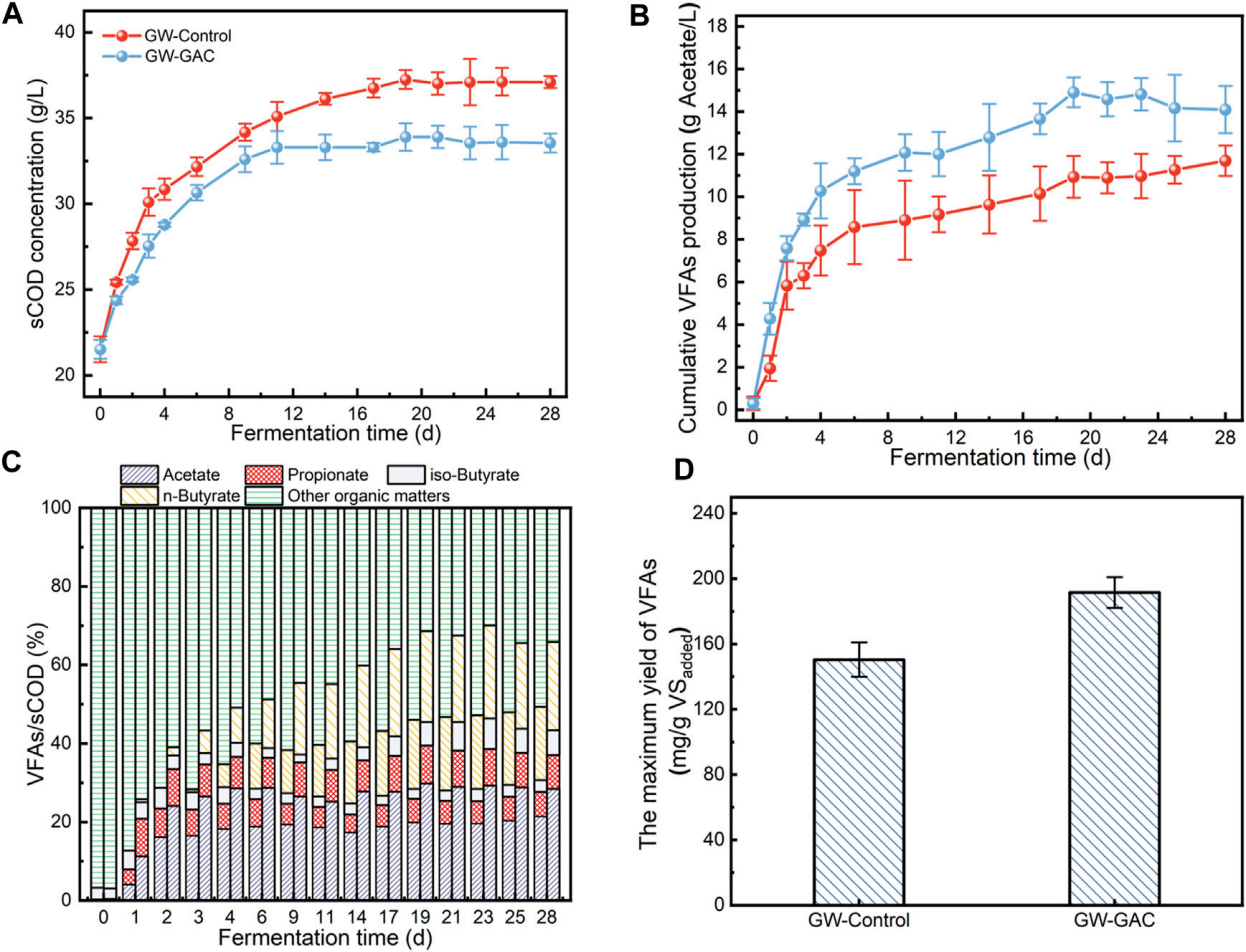
**(A)** sCOD concentration, **(B)** cumulative VFA production (calculated as acetate), **(C)** proportion of VFAs in sCOD (the left column represented the GW-Control reactor, and the right represented the GW-GAC reactor), and **(D)** the maximum yield of VFAs in GW-Control and GW-GAC reactors.

The solubilization of garden waste was expressed in terms of sCOD. Figure 2A shows the sCOD concentration in the control and GAC-amended groups gradually increasing over the initial 14 days and subsequently stabilizing at approximately 3.70 g/L and 3.35 g/L, respectively. In this study, the observed increase in sCOD was attributed to the hydrolysis efficiency of cellulose and hemicellulose present in garden waste biomasses. A lower sCOD level that was observed in the GW-GAC group might be due to the adsorption effect of GAC (Yang et al., 2020b; Zusman et al., 2020), which resulted in a portion of sCOD generated from the hydrolysis of garden waste being adsorbed. Additionally, VFAs could be absorbed by GAC (da Silva and Miranda, 2013). However, in this batch experiment, 50 g/L GAC and 10% TS garden waste were added in a single dose to the GW-GAC reactor, eliminating repeated adsorption/desorption. A more intricate interaction between GAC and VFAs in the anaerobic fermentation system could exist. The enhancing effect of GAC resulted in a faster and higher VFA production (Figure 2B), and quickly saturated by GAC adsorption, thereby achieving a continuous gradual increase in VFA production during subsequent fermentation periods.

The percentage of sCOD from each VFA and other organic matters from both groups is shown in Figure 2C. The VFA/sCOD ratio was a significant indicator, illustrating the extent to which soluble organics could be transformed into VFAs (Fang et al., 2019). Figure 2C shows that after anaerobic fermentation, VFA/sCOD in the GAC-amended reactor surpassed that in the non-amended control reactor. Further calculation revealed that the peak VFAs/sCOD reached 70.01% in the GAC-amended group and only 49.35% in the control group, indicating that the GAC-amended group exhibited a superior acidogenesis capacity. These findings provided additional confirmation that GAC addition enabled efficient hydrolysis and acidogenesis of garden waste.

The change in cumulative VFA production followed a nearly identical trend as that of acetate, and the concentration was higher throughout the whole 28 days in the GAC-amended group (Figure 2B). The results indicated that the addition of GAC may promote electron transfer among fermentative bacteria, specifically enhancing the production of acetate. On the other hand, VFA concentrations in the GW-GAC reactor slightly decreased from day 19. This phenomenon could be attributed to the inhibition of bacterial hydrolysis and acidogenesis activities caused by excessively high VFA concentrations, a phenomenon similar to what was reported by Liang et al. (2023). Such limitations can be overcome if the products were removed from the system continuously (Zhou et al., 2018). Figure 2D shows the maximum total VFA yield in both reactors. The maximum total VFA yield was 191.55 mg/g VS_added_ in the GAC-amended reactor, which increased by 27.35% compared to the control group. The yield of VFAs in this study exceeded the yield reported by Yu et al. (2022), who utilized microwave-assisted ionic liquids as the pretreatment method and then inoculated rumen microbes to ferment wheat stalk, resulting in a VFA yield of 0.180 g/g in a sequencing batch experiment. Therefore, the GAC-amended reactor achieved a high VFA yield during the 28-day anaerobic fermentation period. The notable efficiency in acidogenesis was primarily attributed to that GAC provided a suitable fermentation environment for microbes to produce VFAs and enhanced the mutual metabolism between bacteria. Given the absence of additional enzymes, the VFA production process with GAC amendment offers advantages for garden waste utilization. It minimizes operational costs and enables the direct generation of products without the need for complex pretreatments. As a result, this strategy could provide benefits, such as cost savings, simplified operation, and high yield.

### 3.2 FT-IR analysis of garden waste decomposition

**FIGURE 3:**
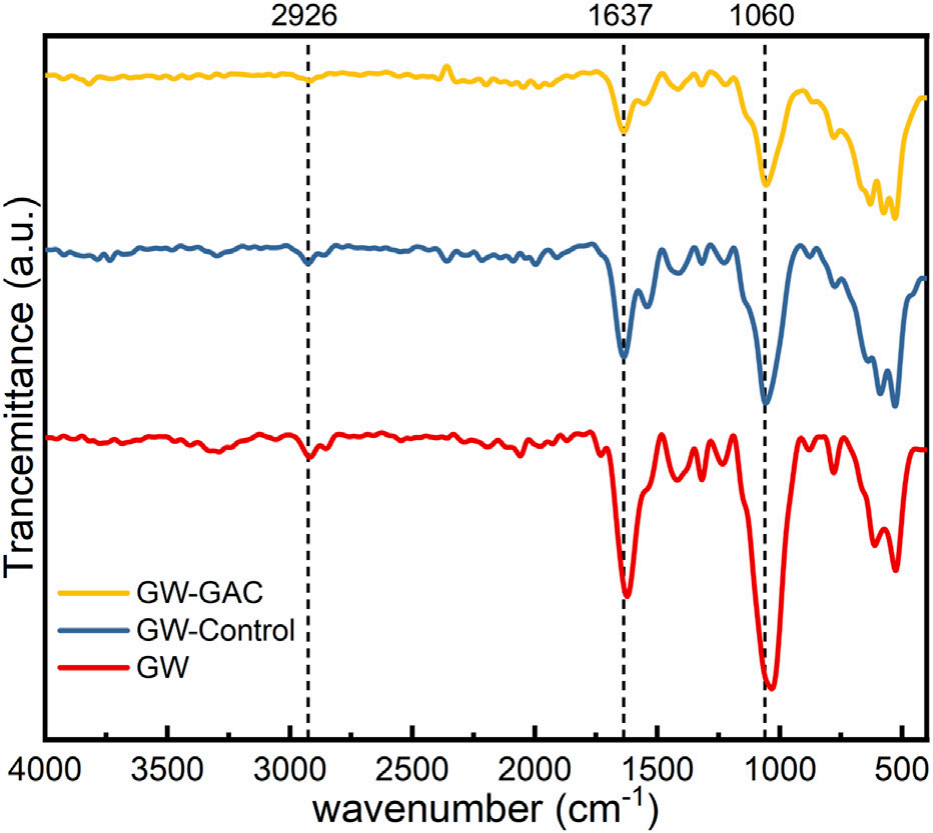
FT-IR spectra of raw garden waste matters in GW-Control and GW-GAC reactors.

FT-IR analysis was utilized to assess the decomposition of garden waste in anaerobic fermentation reactors. Figure 3 shows the FT-IR spectra of the raw garden waste and matters from both reactors at the end of the experiment. While the stretching vibration peak types did not display a noticeable difference between both reactors with or without GAC and the raw garden waste, there was a reduction in peak intensity observed in the presence of GAC, especially for peaks at 2,917 cm^−1^, 1,637 cm^−1^, and 1,060 cm^−1^. The stretching vibration peaks at 2,917 cm^−1^, 1,637 cm^−1^, and 1,060 cm^−1^, represent the saturated C–H stretches related to methyl and methylene groups, C=O side chain stretches, and primary and secondary alcohol (C–O–H) stretches, respectively (Grűbel and Machnicka, 2014; Li et al., 2018b). These peaks are indicative of cellulose and hemicellulose characteristics. All the vibration frequencies corresponding to single-bond stretching and the molecular skeleton are within the 910–1,300 cm^−1^ regions (Abidi et al., 2014).

Figure 3 illustrates the gradual weakening of characteristic peaks in raw garden waste, non-GAC, and GAC-amended groups, indicating the cellulose and hemicellulose degradation of garden waste in both reactors during anaerobic fermentation. Notably, GAC dosing exhibited a more pronounced effect on garden waste degradation, aligning with the cumulative VFA production results (Figure 2B).

### 3.3 Microbial community structure

The VFA production performances were closely correlated with the microbial community structure within the fermentation systems (Li et al., 2018c). In this study, we employed 16S rRNA gene amplicon sequencing to assess the microbial community in both groups and any changes that occurred in their compositions at the phylum and genus levels.

Bacterial alpha diversity was quantified using metrics such as the number of ASVs, Chao1, Shannon diversity index, and Simpson diversity index (Supplementary Table S1). The GW-Control sample had higher Chaos and Shannon indices, and a lower Simpson index compared to the GW-GAC sample, representing that the GW-Control group had a higher richness of microbial community and diversity of microbial community compared to the GW-GAC group (Li et al., 2018a). The results are attributed to the addition of GAC that helps enrich the reactor with more functional bacteria, and some bacteria that were not adapted to high VFA concentrations were eliminated.

**FIGURE 4:**
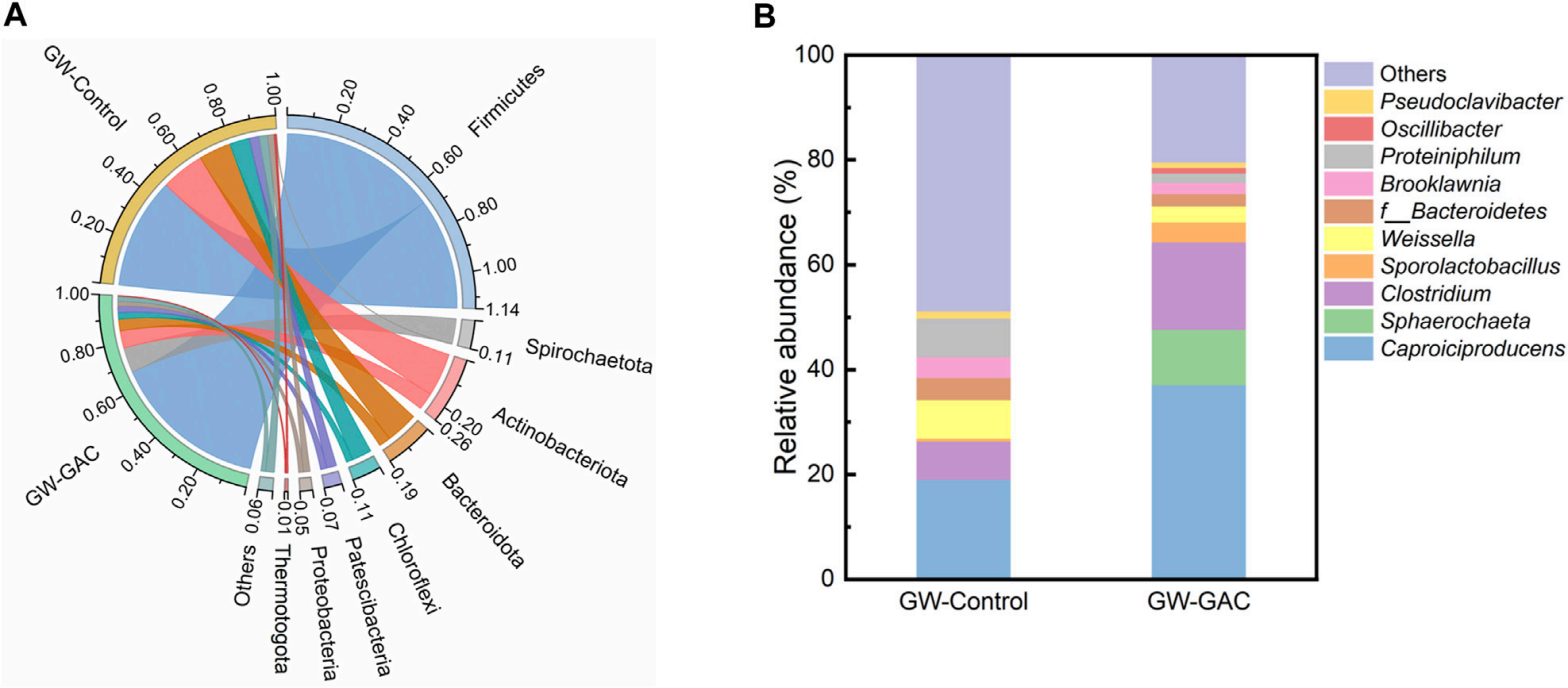
RA of the microbial community during anaerobic fermentation in GW-Control and GW-GAC groups. **(A)** phylum level and **(B)** genus level.

Figure 4A shows the relative abundance (RA) of the eight major phyla, each with a contribution of over 0.50% to the total bacterial sequences in both fermentation systems. In the GW-Control group, the top four phyla were Firmicutes, Actinobacteriota, Bacteroidota, and Chloroflexi, constituting approximately 88.19% of the total abundance. In the GW-GAC group, the predominant phyla were Firmicutes, Spirochaetota, Actinobacteriota, and Bacteroidota, with a combined abundance of approximately 89.96%. These bacteria have the ability to break down complex polymeric organic compounds and ferment monomeric sugars in anaerobic fermentation systems (Zhao et al., 2020). Firmicutes and Spirochaetota, constituting 66.88% and 10.65% of the total, respectively, were the predominant bacteria in the GW-GAC group. These proportions were higher compared to the control group, which had 47.47% of Firmicutes and 0.30% of Spirochaetota. The RA of Actinobacteriota, Bacteroidota, and Chloroflexi was lower in the GW-GAC reactor (7.41%, 5.02%, and 2.78%, respectively) compared to the GW-Control reactor (18.50%, 13.76%, and 8.47%, respectively). These results suggested that GAC might influence the activity of these phyla and lead to alterations in the microbial structure.

Various acidogenic bacteria belonging to the phylum of Firmicutes were well-known to produce proteases, lipases, and various extracellular enzymes to biodegrade multiple complex organics (like proteins and carbohydrates) to VFAs (Chen et al., 2017; Wang et al., 2019). The phylum Spirochaetota is a type of acidogenic bacterium capable of converting carbohydrates into simple VFAs (Yang et al., 2020a). The substantial increase in Firmicutes and Spirochaetota in the GW-GAC reactor suggested that GAC addition promoted the acidification process, leading to a higher VFA concentration in the GW-GAC group. Actinobacteriota can produce hydrolytic enzymes or organic acid and help degrade organic matters (Jang et al., 2014). Relevant studies have shown that Bacteroidota can produce various lytic enzymes and degrade glucose, cellobiose, and amino acids to VFAs, CO_2_, and H_2_ during the degradation of organic materials, respectively (Dykstra and Pavlostathis, 2017; Ros et al., 2017). The phylum Chloroflexi can utilize glucose and VFAs (Ros et al., 2017). A lower RA value of Chloroflexi in the GW-GAC reactor (2.78%) compared to the GW-Control reactor (8.47%) indicated that GAC addition might suppress the activity of this phylum and be conducive to the VFA accumulation. Based on the above analysis, it can be inferred that the accumulation of VFAs in the GW-GAC group may be related to the increase in the RA of Firmicutes and the decrease in the RA of Chloroflexi.

Figure 4B shows the top 10 genera abundance in the GW-GAC fermentation system. Three genera—*Caproiciproducens*, *Clostridium* and *Sporolactobacillus*, all belonging to the phylum Firmicutes—predominated in the GW-GAC group. The genus *Caproiciproducens* had an RA of 36.99% in the GW-GAC group, almost twice that of the GW-Control group (18.95%). It was reported that the primary metabolites of organic acids, including acetate, butyrate, and caproate, can be produced by species within the genus *Caproiciproducens* (Kim et al., 2015). We have not detected the presence of caproate during the tests, thereby suggesting a potential inhibition of caproate production under the condition of pH 5, which is similar to the observation reported by Feng et al. (2018).

The RA value of *Clostridium* was 7.33% in the GW-Control group and 16.68% in the GW-GAC group, and *Clostridium* was identified as the dominant genus in the anaerobic fermentation system and played a crucial role in the transformation of organics to VFAs (Shen et al., 2014). The RA of *Clostridium_sensu_stricto_12* was found to be 2.57% and 3.61% in the GW-Control and GW-GAC groups, respectively. It was reported that *Clostridium_sensu_stricto_12* was a butyrate-producing bacterium with transferases of acetyl-coenzyme and butyrate kinase. These enzymes facilitate the generation of butyrate through the conversion of intracellular and extracellular acetate (Ma et al., 2017; Wang et al., 2020; Li et al., 2022a). This may explain the higher butyrate concentration observed in the GW-GAC group (Figure 1B). RA of *Sphaerochaeta* in the GW-GAC group was 10.59%, whereas it disappeared in the other group. *Sphaerochaeta* belonging to the phylum Spirochaetota had the ability of producing organic acids (Wang et al., 2023). Ritalahti et al. (2012) conducted a study using glucose as substrates in the pure cultures, and it was observed that *Sphaerochaeta* species exhibited the ability to convert soluble Fe (III) oxides into Fe (II). Although their capability of electron transfer to carbon-based conductive materials had not been assessed, they exhibit significant potential of stimulating electron transfer between bacteria (Li et al., 2020). Remarkably, in the presence of GAC, the abundance of *Sphaerochaeta* increased sharply (Figure 4B), suggesting that GAC stimulated their growth and increased the VFA production conversion rate of garden waste. Furthermore, *Oscillibacter*, capable of producing organic acids through indirect extracellular electron transfer using hydrogen as an electron mediator (Ma et al., 2022), was exclusively identified in the GW-GAC group at a proportion of 1.08%, indicating a promoted growth and enrichment of *Oscillibacter* by the addition of GAC. Therefore, hydrogen would stimulate the enrichment of *Oscillibacter* (Dessì et al., 2021), which was corresponding to the results in Figure 1C.

### 3.4 Metagenomics analysis

There was no significant difference in metabolic function between GW-Control and GW-GAC groups in terms of level 2 and 3 metabolic function categories (Supplementary Figure S2). Metabolism, genetic information processing, environmental information processing, and cellular process were the four main metabolic pathways identified in each group. In GW-Control, the primary secondary metabolic pathways were distributed as follows: global and overview maps (28.81%), carbohydrate metabolism (9.00%), amino acid metabolism (7.48%), and energy metabolism (7.49%). Similarly, in GW-GAC, the proportions of these pathways were 28.36%, 9.06%, 7.46%, and 6.96%, respectively (Supplementary Figure S2A). The main metabolic functions of carbohydrate metabolism were glycolysis/gluconeogenesis, amino sugar, and nucleotide sugar metabolism and pyruvate metabolism in both groups (Supplementary Figure S2B), which played a dominant role in the hydrolysis process, providing sufficient available substrates for subsequent acidogenesis.

CAZymes have the function of breaking down the complex carboxylates. All the detected genes coding for CAZymes were further assigned to seven functional classes (Table 2): glycoside hydrolases (GHs), glycosyltransferases (GTs), polysaccharide lyases (PLs), carbohydrate esterases (CEs), carbohydrate-binding modules (CBMs), and auxiliary activity enzymes (AAs). It was apparent that GTs and GHs were the main abundant enzymes, representing the majority of all the CAZyme genes. On the contrary, PLs and SLHs were very scanty in the community. It was worth noting that the abundance of GHs in the GW-GAC reactor showed the highest increase rate (34.88%) compared to the GW-Control reactor.

**TABLE 2:** Abundance of seven functional classes of CAZymes in GW-Control and GW-GAC groups.

Class	GW-Control	GW-GAC
AAs	102582	112656
CBMs	40814	38552
CEs	204384	258864
GHs	674000	909094
GTs	926132	959574
PLs	33906	34896
SLHs	320	236

Importantly, GH families, such as GH5, GH8, and GH51, are known for their versatile functions and ability to hydrolyze both cellulose and hemicellulose, which is dependent on the specific subfamily (Lombard et al., 2014; Peng et al., 2021). Major bacterial cellulases include GH5 and GH9. GH10, GH11, GH26, and GH43 belong to hemicellulases (Peng et al., 2021). The abundance is shown in Supplementary Table S2, and we can dedicate that the addition of GAC promoted the degradation of garden waste from the higher abundance of GHs in the GW-GAC reactor.

**FIGURE 5:**
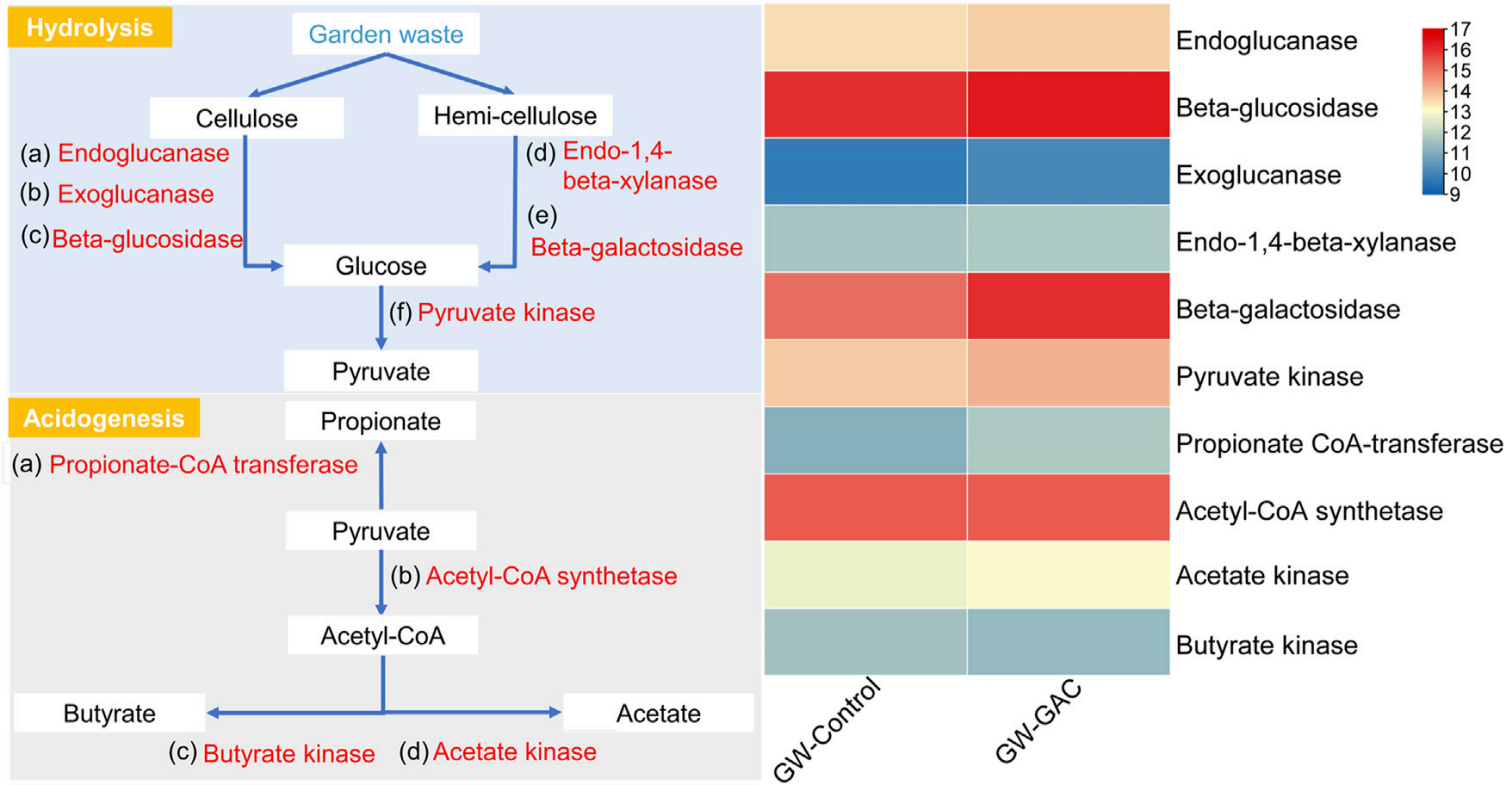
Principal metabolic pathways and a heatmap displaying the abundance of key enzymes related to hydrolysis and acidogenesis in the GW-Control and GW-GAC reactors, with abundance represented by log_2_ values.

To accurately depict the principal metabolic pathways in both non-GAC and GAC-amended groups, the functional metabolic analyses specific to hydrolysis and acidogenesis of each group were identified based on the metagenomics information and KEGG metabolic pathways. Figure 5 shows the principle metabolic pathways and a heatmap based on the abundance of key enzymes related to hydrolysis and acidogenesis in the two groups (Li et al., 2022b). Specific data on key enzymes-encoding genes related to hydrolysis and acidogenesis at each group are shown in supplementary (Supplementary Table S3).

Exoglucanase acts progressively from the ends of the cellulose chains, and endoglucanase has the ability to break down cellulose polymers into their respective reduced sugar and polysaccharide components, with beta-glucosidase (*bglB*) facilitating the final stage of cellulose hydrolysis. Endo-1,4-beta-xylanase, one type of hydrolases, can depolymerize xylan, which is a plant cell component (Tiwari et al., 2023; Tong et al., 2023). The abundances of the above enzymes were higher in the GW-GAC group (Figure 5), which demonstrated that bacteria capable of degrading cellulose and hemicellulose to monosaccharide in the GAC-amended reactor were more abundant. RA of beta-galactosidase (*lacZ*) (4.04‰) in the GW-GAC group was nearly twice as much as that in the GW-Control group (2.38‰). The results above explained the high hydrolysis efficiency of cellulose and hemicellulose. Subsequently, glucose was hydrolyzed to pyruvate, which was further transformed into acetate, propionate, and butyrate. In the GW-GAC group, there was a higher abundance of pyruvate kinase (*pyk*), which is associated with pyruvate formation. Additionally, functional enzymes related to acetate, propionate, and butyrate formation were identified in both fermentation reactors. RA of acetate kinase (*ackA*) related to acetate formation was higher compared to that of propionate CoA-transferase (*pct*) and butyrate kinase (*buk*) related to propionate and butyrate formation. This corresponded to the significantly higher acetate concentration compared to propionate and butyrate in this study (Figure 1B). These results indicated that parts of functional enzymes associated with hydrolysis and acidogenesis were promoted by GAC addition during the fermentation of garden waste.

**FIGURE 6:**
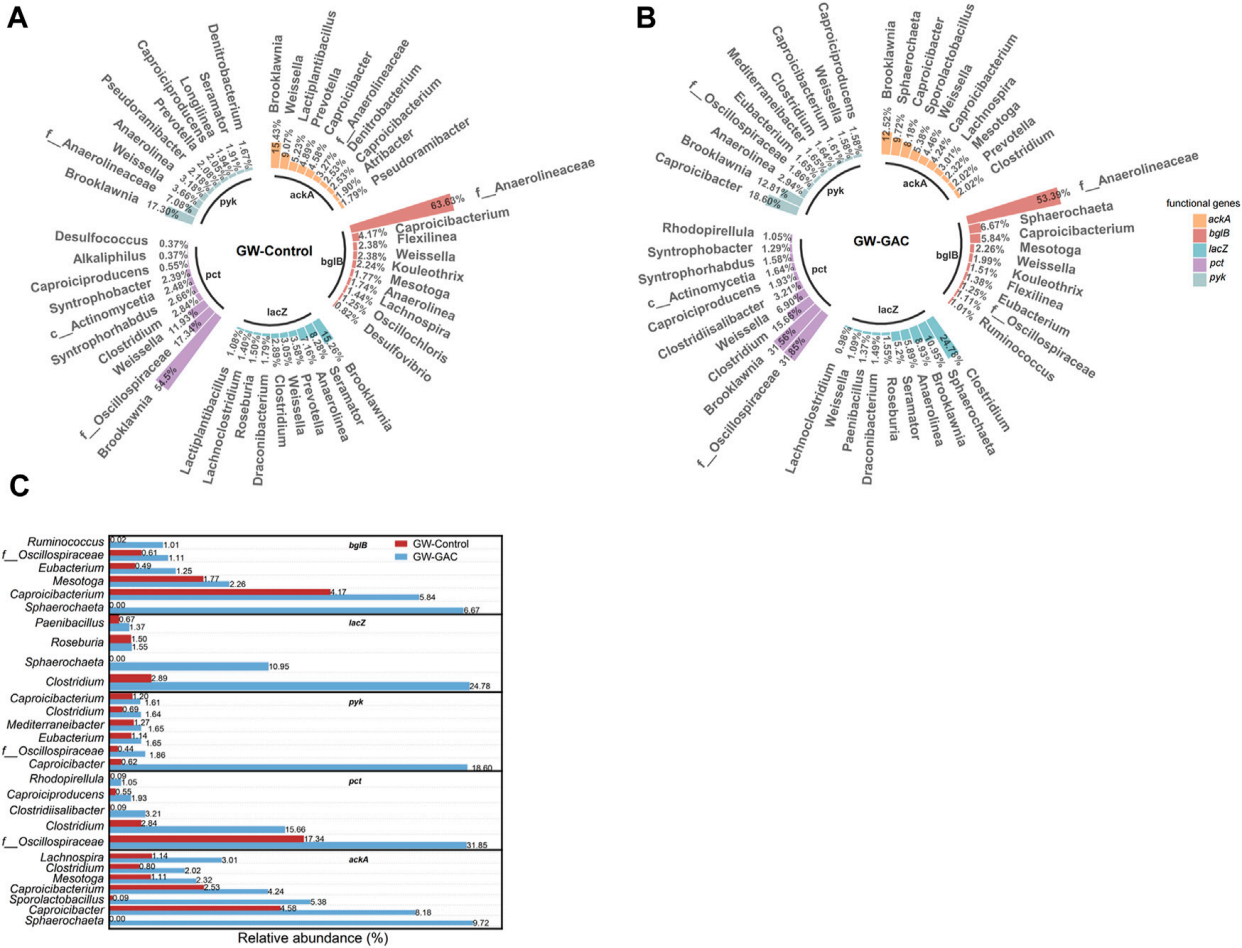
Major genera contributing to the gene abundance of anaerobic fermentation in each group. RA of the top 10 genera in the ranking of gene contribution for the five functional genes (*bglB*, *lacZ*, *pyk*, *pct*, and *ackA*) based on reads_number in the **(A)** GW-Control group, **(B)** GW-GAC group, and **(C)** the difference of main genera in the contribution of the five functional genes between the GW-Control and GW-GAC groups.

To thoroughly investigate the metabolic response of hydrolysis and acidogenesis of garden waste with and without GAC addition, RA values of major genera contributing to the five functional genes (*bglB*, *lacZ*, *pyk*, *pct*, and *ackA*) in both groups were evaluated based on metagenomics analysis. Figure 6A shows that in terms of *bglB* in the GW-Control group, the top three genera were *f__Anaerolineaceae*, *Caproicibacterium* and *Flexilinea*; *Brooklawnia*, *Seramator*, and *Anaerolinea* for *lacZ*; *Brooklawnia*, *f__Anaerolineaceae*, and *Weissella* for *pyk*; *Brooklawnia*, *f__Oscillospiraceae*, and *Weissella* for *pct*; and *Brooklawnia*, *Weissella* and *Lactiplantibacillus* for *ackA*. In the GW-GAC group, the top three genera were changed as follows: *f__Anaerolineaceae*, *Sphaerochaeta*, and *Caproicibacterium* for *bglB*; *Clostridium*, *Sphaerochaeta*, and *Brooklawnia* for *lacZ*; *Caproicibacter*, *Brooklawnia*, and *Anaerolinea* for *pyk*; *f__Oscillospiraceae*, *Brooklawnia*, and *Clostridium* for *pct*; and *Brooklawnia*, *Sphaerochaeta*, and *Caproicibacter* for *ackA* (Figure 6B). Figure 6C depicts the RA of bacteria with increased gene contribution after the addition of GAC. *Sphaerochaeta* exhibited a significant increase in gene contribution to functional genes, reaching 6.67%, 10.95%, and 9.72% for *bglB*, *lacZ*, *and ackA*, respectively. The contributions of *Clostridium* for *lacZ* and *pct* were increased by 757.44% and 450.74%, respectively. The contribution of *Caproicibacter* for *pyk* and *ackA* was apparently improved from 0.62% to 18.60% and from 4.58% to 8.18%, respectively. The results above may explain the higher VFA production observed in the GW-GAC group. Since the abundance of these five functional genes was higher in the GW-GAC group (Figure 5), they contributed to the improvement of hydrolysis and acidogenesis efficiency.

## 4 Conclusion

In this study, GAC was used to promote the VFA production of the garden waste. Acetate, propionate, and butyrate were the main VFA components, which hold significant potential for broad utilization across various industries, potentially substituting fossil fuels in the future. The experimental results showed that GAC addition can achieve a high VFA conversion rate and effectively increase the yield of VFAs with the maximum yield of 191.55 mg/g VS_added_ in the anaerobic fermentation system. In addition, the enrichment of phylum Firmicutes and its genera *Caproiciproducens* and *Clostridium* was identified as the key factor contributing to the substantial increase of VFA production in the GAC-amended fermentation system, and the GAC-amended reactor exhibited a higher RA of *Sphaerochaeta* and *Oscillibacter* that are capable of electron transfer. Furthermore, by metagenomics analyzing, GAC addition improved the abundance of key functional enzymes related to hydrolysis and acidogenesis. Furthermore, the main contributors of upregulated genes (*bglB*, *lacZ*, *pyk*, *pct*, and *ackA*) were *Sphaerochaeta*, *Clostridium*, and *Caproicibacter*. In summary, the garden waste can be used as the feedstock of efficient VFA production by addition GAC without any complex pretreatments and extra enzymes, which offers a novel perspective on the anaerobic fermentation of lignocellulosic biomass for VFA production, potentially leading to cost savings for energy recovery from lignocellulose and simplification of the treatment process.

## Data Availability

The data presented in the study are deposited in theNCBI Sequence Read Archive repository https://www.ncbi.nlm.nih.gov/ accession number: SRP469041, SRP469105.

## References

[B1] Abidi N. Cabrales L. Haigler C. H. (2014). Changes in the cell wall and cellulose content of developing cotton fibers investigated by FTIR spectroscopy. Carbohydr. Polym. 100, 9–16. 10.1016/j.carbpol.2013.01.074 24188832

[B2] APHA (2005). Standard methods for water and wastewater examination, 21st. Washington DC, USA: American Public Health Association.

[B3] Cao W. Fang S. Wu Q. Wu Y. Feng L. Xie Z. (2023). Choline chloride pretreatment on volatile fatty acids promotion from sludge anaerobic fermentation: in-situ deep eutectic solvents-like formation for EPS disintegration and associated microbial functional profiles upregulation. Chem. Eng. J. 467, 143556. 10.1016/j.cej.2023.143556

[B4] Chen Y. Jiang X. Xiao K. Shen N. Zeng R. J. Zhou Y. (2017). Enhanced volatile fatty acids (VFAs) production in a thermophilic fermenter with stepwise pH increase–Investigation on dissolved organic matter transformation and microbial community shift. Water Res. 112, 261–268. 10.1016/j.watres.2017.01.067 28178608

[B5] Chidthaisong A. Conrad R. (2000). Specificity of chloroform, 2-bromoethanesulfonate and fluoroacetate to inhibit methanogenesis and other anaerobic processes in anoxic rice field soil. Soil Biol. Biochem. 32 (7), 977–988. 10.1016/S0038-0717(00)00006-7

[B6] Darvekar P. Liang C. Karim M. N. Holtzapple M. T. (2019). Effect of headspace gas composition on carboxylates production in open-culture fermentation of corn stover. Biomass Bioenergy 126, 57–61. 10.1016/j.biombioe.2019.04.019

[B7] da Silva A. H. Miranda E. A. (2013). Adsorption/desorption of organic acids onto different adsorbents for their recovery from fermentation broths. J. Chem. Eng. Data 58 (6), 1454–1463. 10.1021/je3008759

[B8] Dessì P. Sánchez C. Mills S. Cocco F. G. Isipato M. Ijaz U. Z. (2021). Carboxylic acids production and electrosynthetic microbial community evolution under different CO_2_ feeding regimens. Bioelectrochemistry 137, 107686. 10.1016/j.bioelechem.2020.107686 33142136

[B9] Dykstra C. M. Pavlostathis S. G. (2017). Methanogenic biocathode microbial community development and the role of bacteria. Environ. Sci. Technol. 51 (9), 5306–5316. 10.1021/acs.est.6b04112 28368570

[B10] Fang S. Cao W. Wu Q. Cheng S. Yang Y. Liu J. (2023). Multifaceted roles of methylisothiazolinone intervention in sludge disintegration and acidogenic and methanogenic pathways for efficient carboxylate production during anaerobic fermentation. Chem. Eng. J. 472, 145022. 10.1016/j.cej.2023.145022

[B11] Fang W. Zhang P. Zhang T. Requeson D. C. Poser M. (2019). Upgrading volatile fatty acids production through anaerobic co-fermentation of mushroom residue and sewage sludge: performance evaluation and kinetic analysis. J. Environ. Manag. 241, 612–618. 10.1016/j.jenvman.2019.02.052 30962005

[B12] Feng K. Li H. Zheng C. (2018). Shifting product spectrum by pH adjustment during long-term continuous anaerobic fermentation of food waste. Bioresour. Technol. 270, 180–188. 10.1016/j.biortech.2018.09.035 30218934

[B13] Fonoll X. Shrestha S. Khanal S. K. Dosta J. Mata-Alvarez J. Raskin L. (2021). Understanding the anaerobic digestibility of lignocellulosic substrates using rumen content as a cosubstrate and an inoculum. ACS ES&T Eng. 1 (3), 424–435. 10.1021/acsestengg.0c00164

[B14] Grűbel K. Machnicka A. (2014). Infrared wave analysis after hydrodynamic and acoustic cavitation as effective method of confirming sewage sludge destruction. J. Environ. Sci. Health, Part A. 49 (1), 101–107. 10.1080/10934529.2013.824738 24117089

[B15] Huang Y. Cai B. Dong H. Li H. Yuan J. Xu H. (2022). Enhancing anaerobic digestion of food waste with granular activated carbon immobilized with riboflavin. Sci. Total Environ. 851, 158172. 10.1016/j.scitotenv.2022.158172 35988634

[B16] Jang H. M. Cho H. U. Park S. K. Ha J. H. Park J. M. (2014). Influence of thermophilic aerobic digestion as a sludge pre-treatment and solids retention time of mesophilic anaerobic digestion on the methane production, sludge digestion and microbial communities in a sequential digestion process. Water Res. 48, 1–14. 10.1016/j.watres.2013.06.041 23871253

[B17] Kim B. C. Jeon B. S. Kim S. Kim H. Um Y. Sang B. I. (2015). *Caproiciproducens galactitolivorans* gen. nov., sp nov., a bacterium capable of producing caproic acid from galactitol, isolated from a wastewater treatment plant. Int. J. Syst. Evol. Microbiol. 65, 4902–4908. 10.1099/ijsem.0.000665 26474980

[B18] Kumar R. Kim T. H. Basak B. Patil S. M. Kim H. H. Ahn Y. (2022). Emerging approaches in lignocellulosic biomass pretreatment and anaerobic bioprocesses for sustainable biofuels production. J. Clean. Prod. 333, 130180. 10.1016/j.jclepro.2021.130180

[B19] Li B.-Y. Xia Z.-Y. Gou M. Sun Z.-Y. Huang Y.-L. Jiao S.-B. (2022a). Production of volatile fatty acid from fruit waste by anaerobic digestion at high organic loading rates: performance and microbial community characteristics. Bioresour. Technol. 346, 126648. 10.1016/j.biortech.2021.126648 34974105

[B20] Li L. He J. Wang M. Xin X. Xu J. Zhang J. (2018a). Efficient volatile fatty acids production from waste activated sludge after ferrate pretreatment with alkaline environment and the responding microbial community shift. ACS Sustain. Chem. Eng. 6 (12), 16819–16827. 10.1021/acssuschemeng.8b04115

[B21] Li Q. Gao X. Liu Y. Wang G. Li Y.-Y. Sano D. (2021). Biochar and GAC intensify anaerobic phenol degradation via distinctive adsorption and conductive properties. J. Hazard. Mater. 405, 124183. 10.1016/j.jhazmat.2020.124183 33092879

[B22] Li X. Wei Y. Xu J. Xu N. He Y. (2018b). Quantitative visualization of lignocellulose components in transverse sections of moso bamboo based on FTIR macro- and micro-spectroscopy coupled with chemometrics. Biotechnol. Biofuels 11 (1), 263. 10.1186/s13068-018-1251-4 30263064 PMC6157062

[B23] Li Y. Chen Z. Peng Y. Huang W. Liu J. Mironov V. (2022b). Deeper insights into the effects of substrate to inoculum ratio selection on the relationship of kinetic parameters, microbial communities, and key metabolic pathways during the anaerobic digestion of food waste. Water Res. 217, 118440. 10.1016/j.watres.2022.118440 35429887

[B24] Li Y. Liu M. Che X. Li C. Liang D. Zhou H. (2020). Biochar stimulates growth of novel species capable of direct interspecies electron transfer in anaerobic digestion via ethanol-type fermentation. Environ. Res. 189, 109983. 10.1016/j.envres.2020.109983 32980032

[B25] Li Z. Chen Z. Ye H. Wang Y. Luo W. Chang J.-S. (2018c). Anaerobic co-digestion of sewage sludge and food waste for hydrogen and VFA production with microbial community analysis. Waste Manag. 78, 789–799. 10.1016/j.wasman.2018.06.046 32559971

[B26] Liang J. Zubair M. Chen L. Chang J. Fang W. Nabi M. (2023). Rumen microbe fermentation of corn stalk to produce volatile fatty acids in a semi-continuous reactor. Fuel 350, 128905. 10.1016/j.fuel.2023.128905

[B27] Lombard V. Golaconda Ramulu H. Drula E. Coutinho P. M. Henrissat B. (2014). The carbohydrate-active enzymes database (CAZy) in 2013. Nucleic Acids Res. 42 (D1), D490–D495. 10.1093/nar/gkt1178 24270786 PMC3965031

[B28] Lovley D. R. (2017). Happy together: microbial communities that hook up to swap electrons. ISME J. 11 (2), 327–336. 10.1038/ismej.2016.136 27801905 PMC5270577

[B29] Ma H. Chen X.-Q. Li R. Wang S. Dong J. Ke W. (2017). First-principles modeling of anisotropic anodic dissolution of metals and alloys in corrosive environments. Acta Mater. 130, 137–146. 10.1016/j.actamat.2017.03.027

[B30] Ma J. Wang Z. Li L. Shi Z. Ke S. He Q. (2022). Granular activated carbon stimulated caproate production through chain elongation in fluidized cathode electro-fermentation systems. J. Clean. Prod. 365, 132757. 10.1016/j.jclepro.2022.132757

[B31] Mankar A. R. Pandey A. Modak A. Pant K. K. (2021). Pretreatment of lignocellulosic biomass: a review on recent advances. Bioresour. Technol. 334, 125235. 10.1016/j.biortech.2021.125235 33957458

[B32] Peng X. Wilken S. E. Lankiewicz T. S. Gilmore S. P. Brown J. L. Henske J. K. (2021). Genomic and functional analyses of fungal and bacterial consortia that enable lignocellulose breakdown in goat gut microbiomes. Nat. Microbiol. 6 (4), 499–511. 10.1038/s41564-020-00861-0 33526884 PMC8007473

[B33] Ramos-Suarez M. Zhang Y. Outram V. (2021). Current perspectives on acidogenic fermentation to produce volatile fatty acids from waste. Rev. Environ. Sci. Bio-Technology 20 (2), 439–478. 10.1007/s11157-021-09566-0

[B34] Ramprakash B. Incharoensakdi A. (2022). Dark fermentative hydrogen production from pretreated garden wastes by *Escherichia coli* . Fuel 310, 122217. 10.1016/j.fuel.2021.122217

[B35] Ren N.-Q. Zhao L. Chen C. Guo W.-Q. Cao G.-L. (2016). A review on bioconversion of lignocellulosic biomass to H_2_: key challenges and new insights. Bioresour. Technol. 215, 92–99. 10.1016/j.biortech.2016.03.124 27090403

[B36] Ritalahti K. M. Justicia-Leon S. D. Cusick K. D. Ramos-Hernandez N. Rubin M. Dornbush J. (2012). *Sphaerochaeta globosa* gen. nov., sp nov and *Sphaerochaeta pleomorpha* sp nov., free-living, spherical *spirochaetes* . Int. J. Syst. Evol. Microbiol. 62, 210–216. 10.1099/ijs.0.023986-0 21398503

[B37] Ros M. de Souza Oliveira Filho J. Perez Murcia M. D. Bustamante M. A. Moral R. Coll M. D. (2017). Mesophilic anaerobic digestion of pig slurry and fruit and vegetable waste: dissection of the microbial community structure. J. Clean. Prod. 156, 757–765. 10.1016/j.jclepro.2017.04.110

[B38] Sawatdeenarunat C. Surendra K. C. Takara D. Oechsner H. Khanal S. K. (2015). Anaerobic digestion of lignocellulosic biomass: challenges and opportunities. Bioresour. Technol. 178, 178–186. 10.1016/j.biortech.2014.09.103 25446783

[B39] Shen L. Hu H. Ji H. Cai J. He N. Li Q. (2014). Production of poly (hydroxybutyrate–hydroxyvalerate) from waste organics by the two-stage process: focus on the intermediate volatile fatty acids. Bioresour. Technol. 166, 194–200. 10.1016/j.biortech.2014.05.038 24907579

[B40] Soares A. Kampas P. Maillard S. Wood E. Brigg J. Tillotson M. (2010). Comparison between disintegrated and fermented sewage sludge for production of a carbon source suitable for biological nutrient removal. J. Hazard. Mater. 175(1), 733–739. 10.1016/j.jhazmat.2009.10.070 19932559

[B41] ten Hoeve M. Bruun S. Jensen L. S. Christensen T. H. Scheutz C. (2019). Life cycle assessment of garden waste management options including long-term emissions after land application. Waste Manag. 86, 54–66. 10.1016/j.wasman.2019.01.005 30902240

[B42] Tiwari B. R. Brar S. K. Surampalli R. Y. (2023). Enhancing thermophilic anaerobic digestion of municipal sludge: an investigation. J. Water Process Eng. 56, 104293. 10.1016/j.jwpe.2023.104293

[B43] Tong X. He Z. B. Zheng L. Q. Pande H. Ni Y. H. (2023). Enzymatic treatment processes for the production of cellulose nanomaterials: a review. Carbohydr. Polym. 299, 120199. 10.1016/j.carbpol.2022.120199 36876810

[B44] Wang P. Guo Y. Yu M. Riya S. Zheng Y. Ren L. (2023). The effect and mechanism of polyethylene terephthalate microplastics on anaerobic co-digestion of sewage sludge and food waste. Biochem. Eng. J. 198, 109012. 10.1016/j.bej.2023.109012

[B45] Wang Q. Zhang P. Bao S. Liang J. Wu Y. Chen N. (2020). Chain elongation performances with anaerobic fermentation liquid from sewage sludge with high total solid as electron acceptor. Bioresour. Technol. 306, 123188. 10.1016/j.biortech.2020.123188 32199398

[B46] Wang S. Tao X. Zhang G. Zhang P. Wang H. Ye J. (2019). Benefit of solid-liquid separation on volatile fatty acid production from grass clipping with ultrasound-calcium hydroxide pretreatment. Bioresour. Technol. 274, 97–104. 10.1016/j.biortech.2018.11.072 30502607

[B47] Xu Q. Long S. Liu X. Duan A. Du M. Lu Q. (2023). Insights into the occurrence, fate, impacts, and control of food additives in food waste anaerobic digestion: a Review. Environ. Sci. Technol. 57 (17), 6761–6775. 10.1021/acs.est.2c06345 37070716

[B48] Yang B. Xu H. Liu Y. Li F. Song X. Wang Z. (2020a). Role of GAC-MnO_2_ catalyst for triggering the extracellular electron transfer and boosting CH_4_ production in syntrophic methanogenesis. Chem. Eng. J. 383, 123211. 10.1016/j.cej.2019.123211

[B49] Yang L. Si B. Zhang Y. Watson J. Stablein M. Chen J. (2020b). Continuous treatment of hydrothermal liquefaction wastewater in an anaerobic biofilm reactor: potential role of granular activated carbon. J. Clean. Prod. 276, 122836. 10.1016/j.jclepro.2020.122836

[B50] Yang Y. F. Zhang Y. B. Li Z. Y. Zhao Z. Q. Quan X. Zhao Z. S. (2017). Adding granular activated carbon into anaerobic sludge digestion to promote methane production and sludge decomposition. J. Clean. Prod. 149, 1101–1108. 10.1016/j.jclepro.2017.02.156

[B51] Yu Z. Ma H. Boer E. d. Wu W. Wang Q. Gao M. (2022). Effect of microwave/hydrothermal combined ionic liquid pretreatment on straw: rumen anaerobic fermentation and enzyme hydrolysis. Environ. Res. 205, 112453. 10.1016/j.envres.2021.112453 34843726

[B52] Zhang S. Chang J. L. Lin C. Pan Y. R. Cui K. P. Zhang X. Y. (2017). Enhancement of methanogenesis via direct interspecies electron transfer between *Geobacteraceae* and *Methanosaetaceae* conducted by granular activated carbon. Bioresour. Technol. 245, 132–137. 10.1016/j.biortech.2017.08.111 28892682

[B53] Zhao J. Li Y. Marandola C. Krooneman J. Euverink G. J. W. (2020). Comparison of the microbial communities in anaerobic digesters treating high alkalinity synthetic wastewater at atmospheric and high-pressure (11 bar). Bioresour. Technol. 318, 124101. 10.1016/j.biortech.2020.124101 32947140

[B54] Zhao L. Sun Z.-F. Zhang C.-C. Nan J. Ren N.-Q. Lee D.-J. (2022). Advances in pretreatment of lignocellulosic biomass for bioenergy production: challenges and perspectives. Bioresour. Technol. 343, 126123. 10.1016/j.biortech.2021.126123 34653621

[B55] Zhou M. Yan B. Wong J. W. C. Zhang Y. (2018). Enhanced volatile fatty acids production from anaerobic fermentation of food waste: a mini-review focusing on acidogenic metabolic pathways. Bioresour. Technol. 248, 68–78. 10.1016/j.biortech.2017.06.121 28693950

[B56] Zusman O. B. Kummel M. L. De la Rosa J. M. Mishael Y. G. (2020). Dissolved organic matter adsorption from surface waters by granular composites versus granular activated carbon columns: an applicable approach. Water Res. 181, 115920. 10.1016/j.watres.2020.115920 32505889

