# Supplementary material for: Granular activated carbon enhances volatile fatty acid production in the anaerobic fermentation of garden wastes
Source: Front Bioeng Biotechnol. 2023 Dec 11;11:1330293. doi: 10.3389/fbioe.2023.1330293 (PMC10749581; doi:10.3389/fbioe.2023.1330293)
Supplement: Supplementary file 1 [file DataSheet1.docx]

Supplementary Material

# Metagenomic analysis

**1.1** DNA extraction, library construction, and metagenomic sequencing

Total genomic DNA was extracted from 2 mL sludge samples collected from both reactors at the end of the day using the E.Z.N.A.® Soil DNA Kit (Omega Bio-tek, Norcross, GA, U.S.) according to manufacturer’s instructions. Concentration and purity of extracted DNA was determined with TBS-380 (TurnerBioSystems, USA) and NanoDrop2000 (Thermo, USA), respectively. DNA extract quality was checked on 1% agarose gel.

DNA extract was fragmented to an average size of about 400 bp using Covaris M220 (Gene Company Limited, China) for paired-end library construction. Paired-end library was constructed using NEXTFLEX
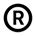
 Rapid DNA-Seq (Bioo Scientific, Austin, TX, USA). Adapters containing the full complement of sequencing primer hybridization sites were ligated to the blunt-end of fragments. Paired-end sequencing was performed on Illumina NovaSeq/Hiseq Xten (Illumina Inc., San Diego, CA, USA) at Majorbio Bio-Pharm Technology Co., Ltd. (Shanghai, China) using NovaSeq Reagent Kits/HiSeq X Reagent Kits according to the manufacturer’s instructions (www.illumina.com).

**1.2 Sequence quality control and genome assembly**

The data were analyzed on the free online platform of Majorbio Cloud Platform (www.majorbio.com). The paired-end Illumina reads were trimmed of adaptors, and low-quality reads (length <50 bp or with a quality value <20 or having N bases) were removed by fastp (Chen et al., 2018) (https://github.com/OpenGene/fastp, version 0.20.0).

**1.3 Gene prediction, taxonomy, and functional annotation**

Open reading frames (ORFs) from each assembled contig were predicted using MetaGene (Noguchi et al., 2006) (http://metagene.cb.k.u-tokyo.ac.jp/). The predicted ORFs with length being or over 100 bp were retrieved and translated into amino acid sequences using the NCBI translation table (http://www.ncbi.nlm.nih.gov/Taxonomy/taxonomyhome.html/index.cgi?chapter=tgencodes#SG1).

A non-redundant gene catalog was constructed using CD-HIT (Fu et al., 2012) (http://www.bioinformatics.org/cd-hit/, version 4.6.1) with 90% sequence identity and 90% coverage. Reads after quality control were mapped to the non-redundant gene catalog with 95% identity using SOAPaligner (Li et al., 2008) (http://soap.genomics.org.cn/, version 2.21), and gene abundance in each sample were evaluated.

Representative sequences of non-redundant gene catalog were aligned to NCBI NR database with e-value cutoff of 1e^-5^ using Diamond (Buchfink et al., 2015) (http://www.diamondsearch.org/index.php, version 0.8.35) for taxonomic annotations. Cluster of orthologous groups of proteins (COG) annotation for the representative sequences was performed using Diamond (Buchfink et al., 2015) (http://www.diamondsearch.org/index.php, version 0.8.35) against eggNOG database with an e-value cutoff of 1e^-5^. The KEGG annotation was conducted using Diamond (Buchfink et al., 2015) (http://www.diamondsearch.org/index.php, version 0.8.35) against the Kyoto Encyclopedia of Genes and Genomes database (http://www.genome.jp/keeg/) with an e-value cutoff of 1e^-5^.

Carbohydrate-active enzymes (CAZymes) annotation was conducted using hmmscan (http://hmmer.janelia.org/search/hmmscan) against CAZy database (http://www.cazy.org/) with an e-value cutoff of 1e^-5^.

# Supplementary Figures and Tables

## Supplementary Figures

**
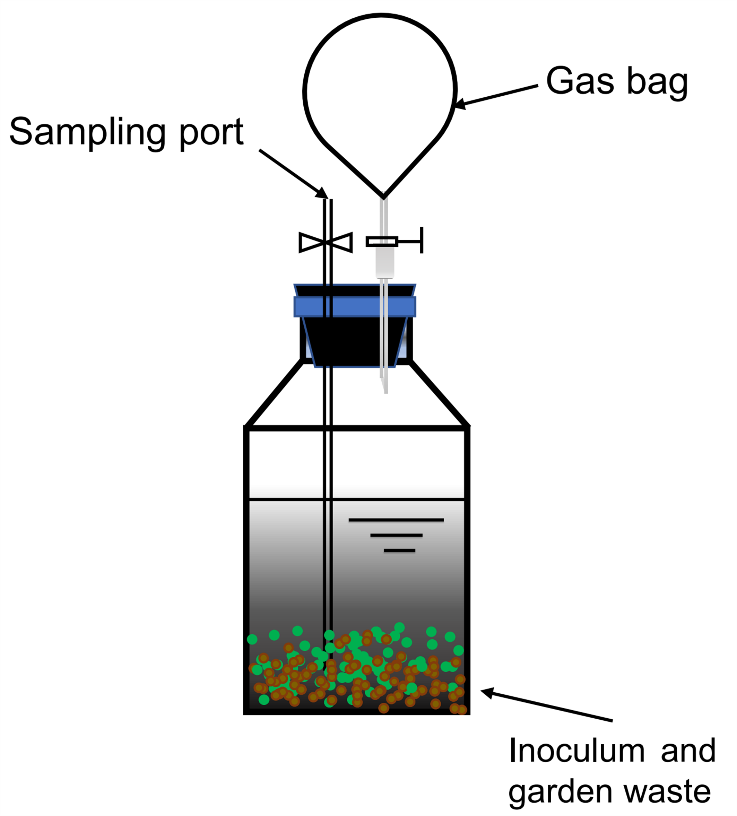
**

**Fig. S1** Schematic diagram of the batch reactor.

**
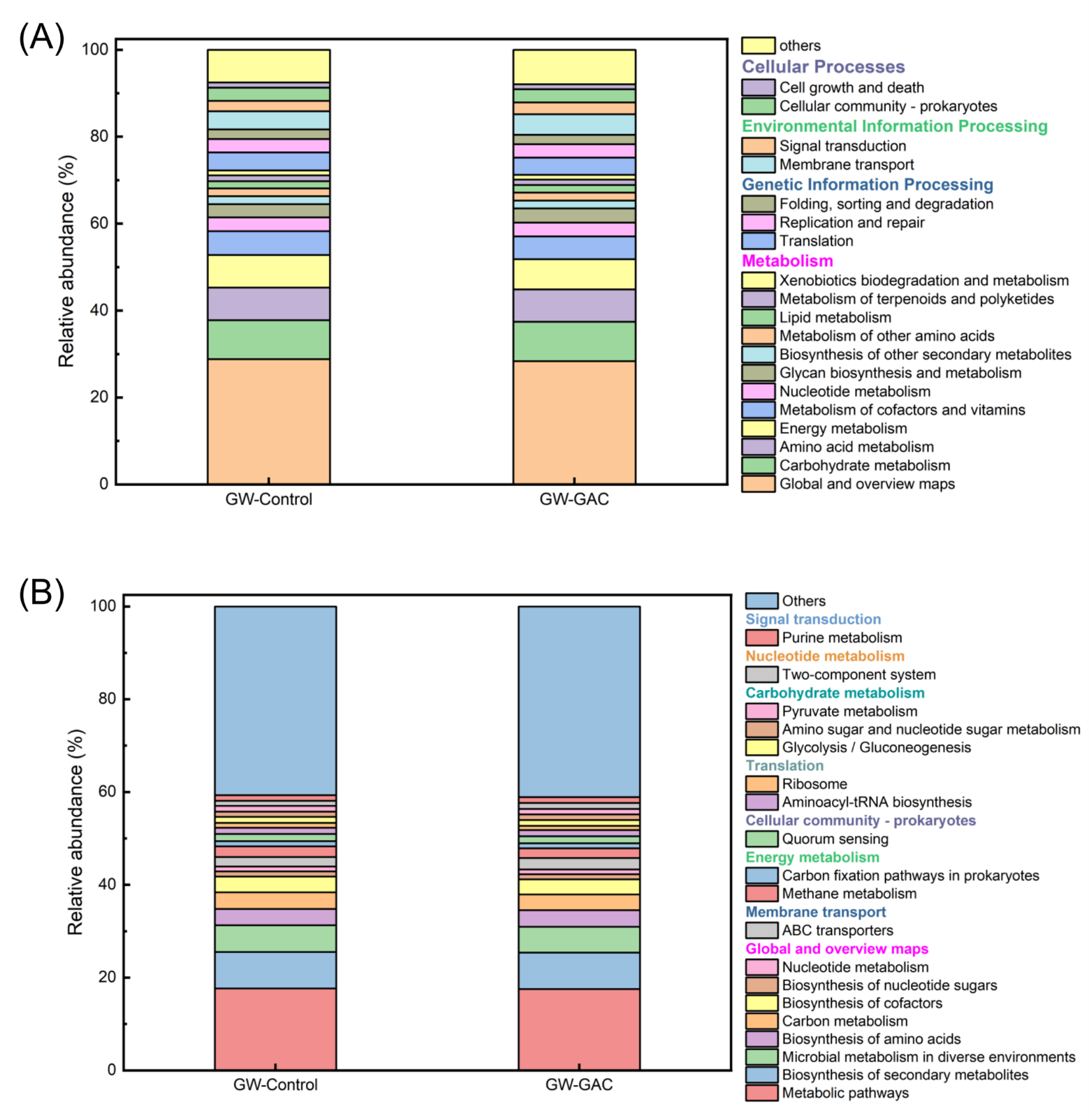
**

**Fig. S2** Microbial metabolism function profiles of each group in AD (RA>1.0%): **(A)** level 2 metabolic function categories. **(B)** level 3 metabolic function categories based on metagenomic analysis.

## Supplementary Tables

**Table S1** Alpha diversity metrics in the two samples on ASV level based on the 16S rRNA analysis.

| Samples | ASVs | Chaos | Shannon | Simpson | Coverage |
| --- | --- | --- | --- | --- | --- |
| GW-Control | 619 | 666.86 | 4.72 | 0.02 | 0.99959 |
| GW-GAC | 530 | 530 | 4.02 | 0.07 | 1 |

**Table S2** The abundance of main functional families of GHs in GW-Control and GW-GAC groups.

| Family | GW-Control | GW-GAC |
| --- | --- | --- |
| GH5 | 4020 | 3706 |
| GH8 | 1872 | 4902 |
| GH9 | 5288 | 7646 |
| GH10 | 3584 | 3942 |
| GH11 | 0 | 20 |
| GH26 | 1316 | 1382 |
| GH43 | 1410 | 2398 |
| GH51 | 11092 | 16356 |

**Table S3** The variations in the abundances of key enzymes-encoding genes related to hydrolysis and acidogenesis at each group based on metagenomic analysis.

| Enzyme | KO | Enzyme number | GW-Control | GW-GAC | GW-Control (‰) | GW-GAC (‰) |
| --- | --- | --- | --- | --- | --- | --- |
| Endoglucanase | K01179 | 3.2.1.4 | 11820 | 13438 | 0.8036 | 0.8014 |
| Beta-glucosidase | K05350 | 3.2.1.21 | 64770 | 83640 | 4.4036 | 4.9879 |
| Exoglucanase | K01225 | 3.2.1.91 | 876 | 1088 | 0.0596 | 0.0649 |
| Endo-1,4-beta -xylanase | K01181 | 3.2.1.8 | 3138 | 3446 | 0.2133 | 0.2055 |
| Beta-galactosidase | K01190 | 3.2.1.23 | 35124 | 67794 | 2.3880 | 4.0429 |
| Pyruvate kinase | K00873 | 2.7.1.40 | 14224 | 18226 | 0.9671 | 1.0869 |
| Propionate CoA- transferase | K01026 | 2.8.3.1 | 2180 | 3422 | 0.1482 | 0.2041 |
| Acetyl-CoA synthetase | K01895 | 6.2.1.1 | 42576 | 42116 | 2.8947 | 2.5116 |
| Acetate kinase | K00925 | 2.7.2.1 | 7040 | 8436 | 0.4786 | 0.5031 |
| Butyrate kinase | K00929 | 2.7.2.7 | 2994 | 2578 | 0.2036 | 0.1537 |

**Reference**

Buchfink, B., Xie, C., and Huson, D.H. (2015). Fast and sensitive protein alignment using DIAMOND. *Nature Methods*. 12(1), 59-60. doi: 10.1038/nmeth.3176.

Chen, S., Zhou, Y., Chen, Y., and Gu, J. (2018). fastp: an ultra-fast all-in-one FASTQ preprocessor. *Bioinformatics*. 34(17), i884-i890. doi: 10.1093/bioinformatics/bty560.

Fu, L., Niu, B., Zhu, Z., Wu, S., and Li, W. (2012). CD-HIT: accelerated for clustering the next-generation sequencing data. *Bioinformatics*. 28(23), 3150-3152. doi: 10.1093/bioinformatics/bts565

Li, R., Li, Y., Kristiansen, K., and Wang, J. (2008). SOAP: short oligonucleotide alignment program. *Bioinformatics*. 24(5), 713-714. doi: 10.1093/bioinformatics/btn025

Noguchi, H., Park, J., and Takagi, T. (2006). MetaGene: prokaryotic gene finding from environmental genome shotgun sequences. *Nucleic Acids Research*. 34(19), 5623-5630. doi: 10.1093/nar/gkl723
